# Dexamethasone Enhances Osteogenic Differentiation of Bone Marrow- and Muscle-Derived Stromal Cells and Augments Ectopic Bone Formation Induced by Bone Morphogenetic Protein-2

**DOI:** 10.1371/journal.pone.0116462

**Published:** 2015-02-06

**Authors:** Masato Yuasa, Tsuyoshi Yamada, Takashi Taniyama, Tomokazu Masaoka, Wei Xuetao, Toshitaka Yoshii, Masaki Horie, Hiroaki Yasuda, Toshimasa Uemura, Atsushi Okawa, Shinichi Sotome

**Affiliations:** 1 Department of Orthopaedic and Spinal Surgery, Graduate School, Tokyo Medical and Dental University, Tokyo, Japan; 2 Global Center of Excellence (GCOE) Program, International Research Center for Molecular Science in Tooth and Bone Diseases, Tokyo Medical and Dental University, Tokyo, Japan; 3 Hyperbaric Medical Center, University Hospital of Medicine, Tokyo Medical and Dental University, Tokyo, Japan; 4 National Institute of Advanced Industrial Science and Technology, Ibaraki, Japan; 5 Department of Orthopaedic Research and Development, Graduate School, Tokyo Medical and Dental University, Tokyo, Japan; Georgia Regents University, UNITED STATES

## Abstract

We evaluated whether dexamethasone augments the osteogenic capability of bone marrow-derived stromal cells (BMSCs) and muscle tissue-derived stromal cells (MuSCs), both of which are thought to contribute to ectopic bone formation induced by bone morphogenetic protein-2 (BMP-2), and determined the underlying mechanisms. Rat BMSCs and MuSCs were cultured in growth media with or without 10-7 M dexamethasone and then differentiated under osteogenic conditions with dexamethasone and BMP-2. The effects of dexamethasone on cell proliferation and osteogenic differentiation, and also on ectopic bone formation induced by BMP-2, were analyzed. Dexamethasone affected not only the proliferation rate but also the subpopulation composition of BMSCs and MuSCs, and subsequently augmented their osteogenic capacity during osteogenic differentiation. During osteogenic induction by BMP-2, dexamethasone also markedly affected cell proliferation in both BMSCs and MuSCs. In an in vivo ectopic bone formation model, bone formation in muscle-implanted scaffolds containing dexamethasone and BMP-2 was more than two fold higher than that in scaffolds containing BMP-2 alone. Our results suggest that dexamethasone potently enhances the osteogenic capability of BMP-2 and may thus decrease the quantity of BMP-2 required for clinical application, thereby reducing the complications caused by excessive doses of BMP-2.

***Highlights:*** 1. Dexamethasone induced selective proliferation of bone marrow- and muscle-derived cells with higher differentiation potential. 2. Dexamethasone enhanced the osteogenic capability of bone marrow- and muscle-derived cells by altering the subpopulation composition. 3. Dexamethasone augmented ectopic bone formation induced by bone morphogenetic protein-2.

## Introduction

Bone grafting is widely used in orthopedic surgery, particularly for the treatment of spinal fusion, complicated fractures, and defects created by tumor resection, all of which require massive bone grafts. Currently, autologous bone grafting is the gold standard for restoration of bone defects because of its superior osteogenic capability, as it provides a source of regenerative cells, an osteoconductive scaffold, and a void filler that biomechanically supports the surrounding bone structure, in contrast to other materials such as allografts and synthetic materials. However, harvesting of autologous bone grafts from patients may cause donor site morbidities such as infection, deep hematoma formation, sensory loss, cosmetic disability, and continuous pain [1–3]. Moreover, the amount of available autologous bone is limited. Many recent studies have focused on developing engineering methods that combine mesenchymal stromal cells (MSCs) with materials to achieve osteogenic induction in vivo. However, these techniques remain unsatisfactory and require improvement.

Bone morphogenetic proteins (BMPs) are members of the TGF-β superfamily [4], and some BMPs have osteoinductive properties. Osteoinduction by decalcified bone extracts was first recognized in the 1960s [5], and the active component for osteoinduction was named BMP, although the responsible proteins were not actually identified. In the 1980s, BMPs were purified, cloned, and synthesized for research use, and numerous studies subsequently applied BMPs for clinical osteoinduction. Among the BMPs, BMP-2 has the strongest osteoinductivity and has been shown to induce differentiation of mesenchymal cells into chondroblasts and osteoblasts [6, 7]. In clinical trials, recombinant human BMP-2 has been shown to accelerate the healing of spinal fusions and open tibial fractures. However, BMP-2 is associated with a high cost, and therefore the amount of BMP that can be feasibly used is low [8, 9]. Furthermore, the use of BMPs is associated with complications, which prevents widespread clinical application [10–22].

Dexamethasone is a synthetic glucocorticoid that has been used clinically as an anti-inflammatory drug, although long-term administration of dexamethasone or other steroids may cause or exacerbate osteoporosis. However, dexamethasone has also been used for decades to differentiate MSCs into adipogenic [23], chondrogenic [24–26], and osteogenic lineages [27–29], although the exact mechanism of how dexamethasone induces differentiation remains unclear. Previously, we hypothesized that dexamethasone does not directly induce MSCs to differentiate into specific lineages but rather augments the responsiveness of MSCs to other differentiation reagents used together with dexamethasone. In particular, we reported that human bone marrow-derived MSCs allowed to proliferate under continuous dexamethasone treatment showed increased osteogenic, adipogenic, and chondrogenic differentiation [29]. It has also been reported that dexamethasone enhances the response of human bone marrow stromal cells to osteogenic stimulation by BMP-2 [30]. However, the mechanism underlying the synergistic effects of dexamethasone and BMP-2 on the osteogenic differentiation of bone marrow stromal cells remains unclear even *in vitro*, and such a synergistic effect has not yet been reported *in vivo*.

We hypothesized that if dexamethasone augments the osteoinductivity of BMPs, the combination of dexamethasone and BMPs would not only augment bone formation but also reduce the required dosage of BMP, thereby reducing the cost and decreasing the complications associated with BMP usage. Thus, the purpose of this study was to determine whether dexamethasone augments bone formation induced by BMP-2. In particular, we investigated the effect of dexamethasone on the osteogenic differentiation of bone marrow-derived stromal cells and muscle-derived stromal cells, both of which are considered to contain subpopulations of osteoprogenitors that contribute to ectopic bone formation induced by BMP-2, and we further determined whether dexamethasone augments heterotopic bone formation induced by BMP-2 in muscle tissue.

## Materials and Methods

### Animals

This study was approved by the Animal Experimentation Committee of Tokyo Medical and Dental University. All animal experiments were performed in accordance with the guidelines of Tokyo Medical and Dental University for the care and use of laboratory animals. Male 7-week-old Fischer344 rats (Sankyo Labo Service Co., Inc., Tokyo, Japan) were used for both in vitro and in vivo experiments. The rats were housed at 22–24°C with a 12-hour light/12-hour dark cycle and a standard chow diet and water provided ad libitum. Animals were sacrificed by exposure to 100% CO_2_ followed by cervical dislocation.

### Cell Isolation and Culture

**1: Bone marrow-derived stromal cells (BMSCs).** BMSCs were obtained from the femurs of rats according to the method described by Maniatopoulos et al. [31]. Briefly, rats were sacrificed by exposure to 100% CO_2_ followed by cervical dislocation. Subsequently, the femurs were dissected by sterilized techniques, the metaphyses were removed, a 21 G needle was inserted into the femur, and the bone marrow was flushed using a 10 ml syringe filled with Dulbecco’s modified Eagle medium (DMEM; Sigma-Aldrich, St. Louis, MO, USA) containing 10% fetal bovine serum (Life Technologies, Carlsbad, CA, USA) until all of the bone marrow was flushed out. The flushed bone marrow cells were then plated in T-75 culture flasks (Becton, Dickinson, and Company, Franklin Lakes, NJ, USA) and cultured in culture medium (DMEM containing 10% fetal bovine serum (Sigma-Aldrich) and 1% antibiotic-antimycotic (100U/ml penicillin G sodium, 100 μg/ml streptomycin sulfate, and 0.25 µg/ml amphotericin B; Life Technologies)] or culture medium supplemented with 10^-7^ M dexamethasone (DEX, Sigma-Aldrich) (DEX medium) at 37°C in a humidified atmosphere with 5% CO_2_. The medium was changed twice a week thereafter. When the attached P0 BMSCs in the cultured flasks became subconfluent (7–10 days), they were reseeded as P1 cultures. The P0 cells and P1 cells were detached using trypsin-EDTA (0.25% trypsin, 1 mM EDTA; Life Technologies) and used for the subsequent experiments. BMSCs cultured in the culture medium alone, i.e., the control medium, are referred to as BM cells, and cells cultured in the DEX medium are referred to as BM-Dex cells hereafter.

**2: Muscle tissue-derived stromal cells (MuSCs).** MuSCs were obtained from rat calf and thigh muscles according to a method described previously [32]. Briefly, after rats were sacrificed as described above, calf and thigh muscle tissue was collected and minced on a dish using sterile techniques and then digested with 0.3% collagenase (Collagenase Type II, Worthington Biochemical Co., NJ, USA) dissolved in DMEM containing 1% antibiotic-antimycotic at 37°C for 90 minutes. After enzymatic digestion, the muscle tissue and released cells were washed twice with PBS and once with the control medium, and then placed into T-75 flasks and cultured in the control medium or DEX medium in a humidified atmosphere with 5% CO_2_. After one week of culture, the cells attached to the flasks and the cells that had proliferated on the suspended muscle fragments were detached using trypsin-EDTA and reseeded with the suspended muscle fragments into T-225 culture flasks (P1). After 7–10 days of culture, the P1 cells were used for the following experiments. Muscle tissue-derived cells cultured in the control medium are referred to as Mu cells and cells cultured in the DEX medium are referred to as Mu-Dex cells hereafter.

### Cell proliferation assay

BMSCs and MuSCs were cultured for 35 days in the control medium or DEX medium (10^-8^, 10^-7^, or 10^-6^ M DEX). Each culture was reseeded when it reached 80–90% confluence, and the cells were counted using an automated cell counter (LUNA^TM^ Automated Cell Counter, Logos Biosystems, Inc., Annandale, VA, USA). Briefly, when the cells were reseeded, an aliquot of cell suspension was mixed with the same volume of 0.4% Trypan blue staining solution, and 10 μl of the mixture was then loaded into a cell counting chamber. The instrument obtained a magnified image of the chamber and analyzed the image to distinguish and count the live cells.

### Osteogenic differentiation

Cells were maintained in osteogenic medium containing 10 mM β-glycerophosphate (Sigma-Aldrich) and 50 μg/ml ascorbic acid phosphate (Wako, Osaka, Japan), with or without 10^-7^ M dexamethasone and with or without 100 ng/ml BMP-2 (E. coli-derived recombinant-human BMP-2, Osteopharma, Osaka, Japan). The treatment groups were defined as (-), AG, AGD, AGB, and AGBD according to the combination of osteogenic supplements used. ‘A’ indicates ascorbic acid phosphate, ‘G’ indicates β-glycerophosphate, ‘B’ indicates BMP-2, and ‘D’ indicates dexamethasone. (-) indicates the absence of supplement treatment. For example, BM-Dex cells differentiated with ascorbic acid phosphate, β-glycerophosphate, and BMP-2 were termed ‘BM-Dex-AGB’.

### Colony formation assay

To investigate the effects of dexamethasone on the subpopulation composition of BMSCs and MuSCs, we conducted a colony formation analysis by seeding the cells at low density to obtain single-cell-derived colonies [33]. BM cells, BM-Dex cells, and Mu-Dex cells at P1 were seeded in 100-mm culture dishes at a density of 5 cells/cm^2^, and Mu cells were seeded at a density of 4 cells/cm^2^ because they proliferated much faster than the other cells. In each group, four dishes were cultured with the control medium and another four dishes were cultured with the DEX medium for 7 days for colony formation. After 7 days of colony formation, each medium was changed to osteogenic medium and the cells were cultured for 7 days to initiate osteogenic differentiation. The dishes were then subjected to ALP staining and imaging using an imaging scanner (GT-X970, Epson, Nagano, Japan), followed by crystal violet staining to count the total number of colonies.

### Osteogenic differentiation assay

The cells of each group at P1 were seeded in 6-well culture plates at a density of 2 × 10^5^/well. The cells were cultured until the wells reached 80% confluence, and the culture medium in each well was then changed to osteogenic medium. At 10 days of differentiation, the cells in each well were subjected to ALP staining, Von Kossa staining, total RNA extraction, or protein extraction for western blot analysis.

### Proliferation assay during differentiation

To assess the effects of osteogenic reagents on cell proliferation, cells were seeded at a density of 1 × 10^4^ cells/well in 6-well culture plates and the medium was changed to osteogenic medium on the next day. The cells were fixed with 10% neutral buffered formalin every three days and stained with crystal violet. After extensive washing with water, the dye bound to the cells was extracted using 2% SDS, and the absorbance at 585 nm was measured using a micro plate reader.

### ALP staining

Cells were fixed with 4% paraformaldehyde for 30 minutes. After washing with PBS, the cells were incubated with a filtered mixture of naphthol AS-MX phosphate (0.1 mg/ml, Sigma-Aldrich), N,N-dimethylformamide (0.5%, Wako), MgCl_2_ (2 mM), and Fast Blue BB salt (0.6 mg/ml, Sigma-Aldrich) in 0.1 M Tris-Cl (pH 8.5) for 30 min at room temperature.

### Von Kossa staining

The mineralization of the cells was assessed by Von Kossa staining. The fixed cells were incubated with 1 ml of 3% silver nitrate solution and incubated for 10 minutes under UV light.

### Gene expression analysis

Total RNA was isolated from culture dishes using RNAiso Plus (Takara Bio Inc., Shiga, Japan) and first-strand cDNA was prepared using the PrimeScript RT reagent kit with gDNA Eraser (Takara Bio Inc.) according to the manufacturers’ instructions. Gene expression was quantified by real-time PCR using a Mx3000P QPCR System (Agilent Technologies, Inc., Santa Clara, CA., USA) and GoTaq qPCR Master Mix (Promega Co., Fitchburg, WI, USA) according to the manufacturers’ instructions. The primer sets used in the study are described in Table 1. Standards were prepared from one specific sample, and every PCR reaction was run with a standard curve to determine the values of the samples relative to those of the standards. YWHAZ was used to normalize the amount of template present in each sample.

**Table 1:** RT-PCR primers used in this study.

**Genes**	**Forward**	**Reverse**
YWHAZ	GAGTCGTACAAAGACAGCACGCTAA	GTGGGACAGCATGGATGACAA
ALP	CACGTTGACTGTGGTTACTGCTGA	CCTTGTAACCAGGCCCGTTG
Osteocalcin	GGTGCAGACCTAGCAGACACCA	AGGTAGCGCCGGAGTCTATTCA

### Western blot analysis

Proteins were extracted from both bone marrow-derived cells and muscle tissue-derived cells. The total cellular protein was prepared by lysing the cells in SDS sample buffer and boiled at 95°C for five minutes, and protein concentrations were determined using the BCA Protein Reagent Kit (Pierce, Rockford, IL, USA). Primary antibodies for P-SMAD1/5 (Ser463/465) (#13820 rabbit mAb, Cell Signaling Technology Inc., Beverly, MA, USA), SMAD1/5/8 (sc-6031-R rabbit mAb, Santa Cruz Biotechnology, Inc, Dallas, TX, USA), and α-tubulin (#2144 rabbit mAb, Cell Signaling Technology, Inc.) were obtained. Proteins (25 μg) were separated by 10% sodium dodecyl sulfate polyacrylamide gel electrophoresis (SDS-PAGE) and then transferred to a polyvinylidene difluoride (PVDF) membrane. After blocking with PVDF Blocking Reagent, the membranes were hybridized with the primary antibody dissolved in Can Get Signal Solution 1 (Toyobo Life Science, Tokyo, Japan) overnight at 4°C and then hybridized with horseradish peroxidase (HRP)-conjugated anti-rabbit immunoglobulin G secondary antibody (sc-2313, Santa Cruz Biotechnology, Inc.) dissolved in Can Get Signal solution 2 (Toyobo Life Science) for 1 h at room temperature. Subsequently, Lumigen TMA-6 (Lumigen Inc., Southfield, MI, USA) was added onto the membrane and allowed to stand for 2 min. Signals were detected by using an enhanced chemiluminescence method (Ez-Capture MG, Atto, Tokyo, Japan).

### Ectopic bone formation study

**1. Ectopic bone formation by bone marrow and muscle tissue-derived cells.** BMSCs and MuSCs were isolated as described above and cultured in culture medium with or without 10^-7^ M dexamethasone and with or without 100 ng/ml BMP-2 for three weeks. The cells were washed well with PBS to clear residual dexamethasone and BMP-2, detached using trypsin-EDTA, seeded in a porous β-tricalcium phosphate block (β-TCP block; 5 mm × 5 mm × 5 mm, porosity: 75%, pore size: 100–200 μm, Olympus Co., Tokyo, Japan) using a method described previously [34], and then subcutaneously transplanted into rats. Briefly, the cells were suspended in rat plasma (10^6^ cells/ ml) prepared by centrifugation of whole blood supplemented with citrate phosphate dextrose for anticoagulation. The cell suspension was mixed with 2% CaCl_2_ solution to initiate fibrin gel formation and then immediately introduced into the β-TCP blocks. Under general anesthesia by intraperitoneal injection of chloral hydrate (0.35 mg/g), the rats were placed in the prone position and the dorsal hair was removed. Two small incisions were made in both sides of the back and subcutaneous pouches were made by spreading the subcutaneous tissue. The blocks were implanted into the pouches. The implants were harvested at 4 weeks after transplantation and analyzed histologically.

**2. Quantum dot labeling of the cells at the transplantation site.** Cell-internalizing quantum dots (i-QDs) were prepared by conjugation of Qdot 655 with an internalizing anti-mortalin monoclonal antibody using an antibody conjugation kit (Q22021MP; Life Technologies) as described previously [35]. Prior to implantation, to label the muscle cells at the implantation site, 25 μl of i-QDs was injected directly into the calf muscle of the rats using a Hamilton syringe. One week after the injection, a porous 5 mm^3^ cubic β-TCP block containing 2 μg of BMP-2 was implanted into the muscle pocket of the injected site. The block was harvested two weeks after implantation, immediately immersed in 4% carboxymethyl cellulose, and rapidly frozen in 2-methylbutane precooled by liquid nitrogen. Frozen sections were prepared using Kawamoto’s method [36] and stained with hematoxylin. A fluorescent microscope was used to detect labeled cells in the bone tissue.

**3. Ectopic bone formation by the combination of DEX and BMP-2.** Dexamethasone dissolved in 100% ethanol (80 μl, concentrations: 0, 10^-7^, 10^-6^, and 10^-5^ M) was infiltrated into a porous 5 mm^3^ cubic β-TCP under sterile conditions and air-dried for 60 min in a clean bench, resulting in β-TCP blocks containing 0, 0.031, 0.31 or 3.1 ng of dexamethasone. Subsequently, recombinant human BMP-2 solution (2 μg, 80 μl) was infiltrated into the β-TCP blocks containing dexamethasone. We confirmed that all of the solution had infiltrated into the scaffolds by checking for residual solution at the bottom of the scaffold. The implants were transplanted into the muscles of rats using a previously described method with some modifications [37]. Briefly, under general anesthesia as described above, one central incision (approximately 2 cm) was made and both sides of the back muscle were exposed. The muscle fascia was cut using a sharp scalpel, and four muscle pouches were created using a hemostat. Subsequently, the prepared blocks were transplanted into the pouches one by one. After transplantation, the rats were housed without any restriction. After 3 weeks, the rats were sacrificed by intraperitoneal injection of excess anesthetic, and the implants were harvested. The harvested implants were fixed with 10% neutral buffered formalin, and decalcified sections were stained with hematoxylin and eosin. Bone formation was confirmed by immunohistochemistry using a primary antibody against osteocalcin (1:5000, OCG3, Takara Bio). Because the mineral densities of the woven bone and β-TCP were very similar, the bone tissue could not be distinguished from the scaffold clearly using micro computed tomography. Therefore, we quantified the bone tissue in the porous β-TCP blocks using histological images [29, 34]. Three sections at equal intervals were prepared from one implant and stained with hematoxylin and eosin. Then, bone tissues were selected manually and bone formation was quantified using imaging software. The bone formation area of one implant was determined from the average value of three sections.

### Statistical analysis

Real-time PCR data were analyzed using a two-way repeated-measures analysis of variance (ANOVA), and multiple comparisons were performed using the Bonferroni correction. The data from the CFU assay were analyzed using Student’s t-test. The bone formation ratio was analyzed using a one-way ANOVA. As a post-hoc test, multiple comparisons between groups were performed using Student’s t-test with the Bonferroni correction.

## Results

### 1. Dexamethasone alters the proliferation of bone marrow-derived and muscle-derived stromal cells

**Figure 1:**
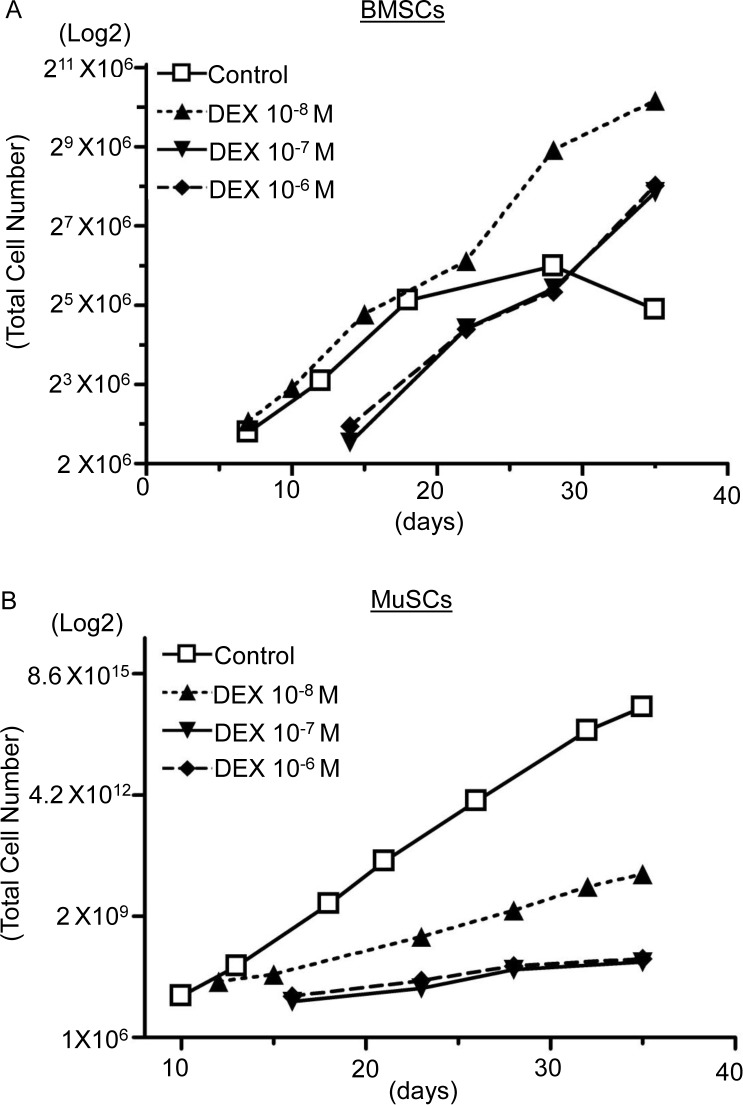
Alteration of proliferation of BMSCs and MuSCs cultured with dexamethasone. A: Proliferation rate of BMSCs cultured with 10^-8^ M, 10^-7^ M, or 10^-6^ M dexamethasone or without dexamethasone for 35 days. Total cell numbers (Y-axis) are expressed using a log2 scale. B: Proliferation rate of MuSCs cultured with 10^-8^ M, 10^-7^ M, or 10^-6^ M dexamethasone or without dexamethasone for 35 days. Total cell numbers (Y-axis) are expressed using a log2 scale.

The BMSCs cultured in the control medium proliferated until 21 days of culture, after which the proliferation rate decreased rapidly. The lowest concentration of dexamethasone (10^-8^M) augmented the proliferation of the BMSCs throughout the experimental period. The proliferation rates of the BMSCs treated with 10^-7^ and 10^-6^ M dexamethasone were lower than those of untreated cells at the early stage of culture. However, the proliferation rates did not decrease during the culture period, in contrast to the proliferation of the untreated BMSCs. Furthermore, the BMSCs treated with 10^-7^ and 10^-6^ M dexamethasone showed similar proliferation (Fig. 1A). MuSCs without dexamethasone treatment proliferated at a much higher rate than dexamethasone-treated MuSCs throughout the culture period. Dexamethasone-treated MuSCs proliferated at much lower rates than cells cultured without dexamethasone. In particular, the proliferation of MuSCs cultured in 10^-7^ and 10^-6^ M dexamethasone was strongly suppressed to similar levels (Fig. 1B). These results indicate that dexamethasone strongly suppresses the proliferation of BMSCs and MuSCs, particularly MuSCs.

### 2. Dexamethasone pretreatment and osteogenic induction by the combination of dexamethasone and BMP-2 enhance the osteogenic differentiation of BMSCs and MuSCs

**Figure 2:**
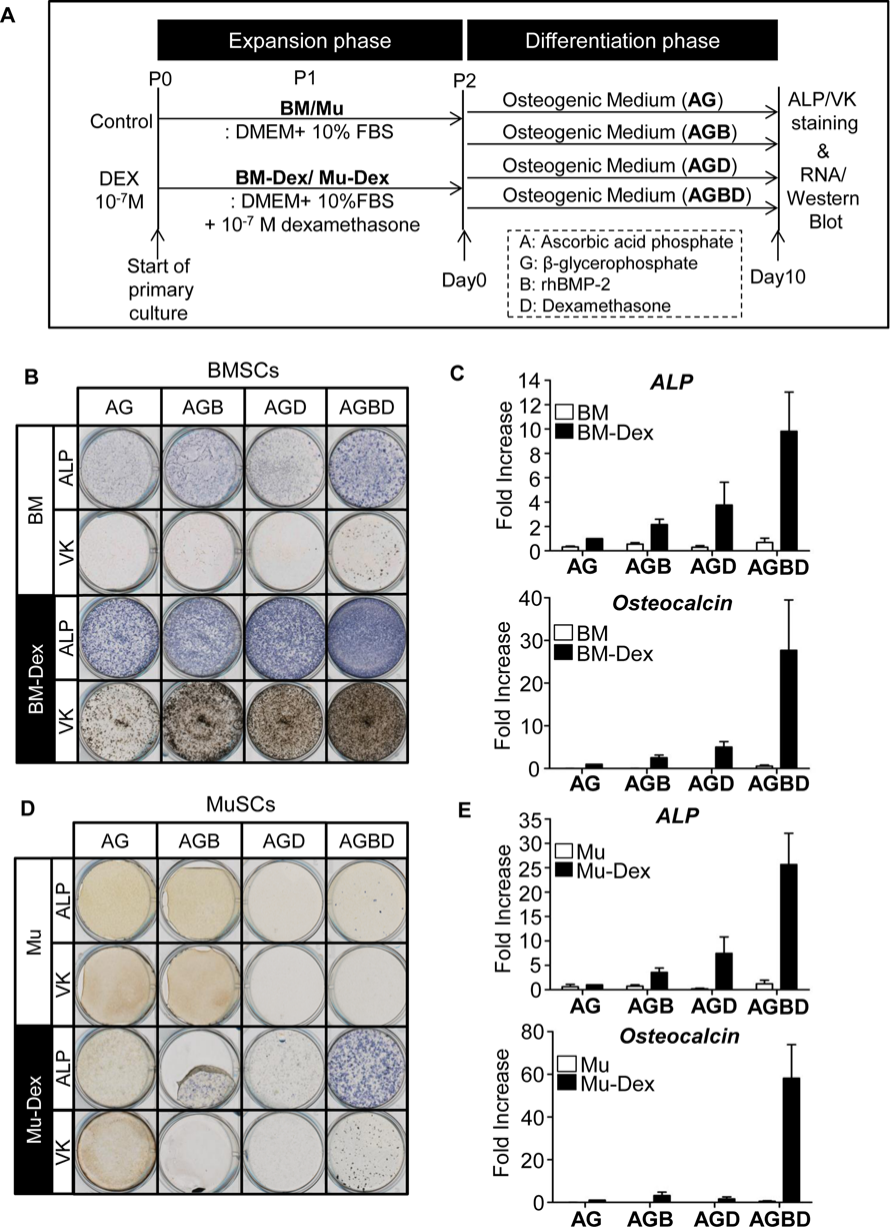
Dexamethasone pretreatment and osteogenic induction with combined dexamethasone and BMP-2 treatment enhance the osteogenic differentiation of BMSCs and MuSCs. A: Schematic representation of the cell culture protocol. Gross images of ALP staining (ALP) and Von Kossa staining (VK) of BMSCs (B) and MuSCs (D). Quantitative analysis of the mRNA expression of ALP and osteocalcin in BMSCs (C) and MuSCs (E). The fold change in gene expression was normalized to that of BM-Dex-AG or Mu-Dex-AG. Bars show the mean and SEM. Statistical significance was confirmed between the BM and BM-Dex groups (ALP: p = 0.015, OCN: p = 0.023) and between the Mu and Mu-DEX groups (ALP: p = 0.019, OCN: p = 0.015). Effects of combinations of differentiation reagents were significant for ALP in BM-Dex (p = 0.032) and Mu-DEX (p = 0.019) and for OCN in Mu-DEX (p = 0.024).

Osteogenic induction was performed as shown in Fig. 2A. Preliminary studies confirmed that in both BMSCs and MuSCs, cells treated with 10^-7^ and 10^-6^ M dexamethasone showed the highest osteogenic differentiation, and there was no significant difference between the concentrations (data not shown). Therefore, in subsequent studies, we compared cells cultured in the control medium and cells cultured in the medium containing 10^-7^ M dexamethasone. ALP staining and Von Kossa staining were used to determine the ALP activity and mineralization capability of the BMSCs (BM and BM-Dex cells) (Fig. 2B), and mRNA expression of osteogenic markers was also evaluated (Fig. 2C). All of these assays showed that the osteogenic capacity of BM-Dex cells was remarkably higher than that of BM cells (*P*<0.05). BM-Dex-AGD cells showed significantly higher ALP and osteocalcin mRNA expression than BM-Dex-AG cells, which were treated with dexamethasone during expansion but not treated with dexamethasone during osteogenic induction. A comparison between BM-Dex-AGB and BM-Dex-AGBD cells revealed that inclusion of dexamethasone during osteogenesis enhanced the effects of BMP-2 on osteogenesis. Among all of the groups, BM-Dex-AGBD cells presented the strongest ALP and Von Kossa staining, with concomitantly high ALP and osteocalcin mRNA expression. The BM-AGBD cells presented the highest ALP and osteocalcin mRNA expression among BM cells, although the expression levels were still far lower than those in BM-Dex cells.

Among Mu cells, although low mRNA expression of ALP was detected, all of the wells were almost negative for ALP staining, and no calcified nodules were detectable on Von Kossa staining (Fig. 2D and 2E). Conversely, the wells containing Mu-Dex cells presented scattered ALP-positive colonies, and the ALP staining intensity of each positive colony was highest in wells containing Mu-Dex-AGBD cells. Von Kossa staining of Mu-Dex-AGBD cells clearly demonstrated mineralized nodules, which confirmed that Mu-Dex cells are capable of mineralization. The scattered nature of the staining indicated that cells with osteogenic capability comprised only a small portion of the Mu-Dex cells. ALP mRNA expression was significantly higher in Mu-Dex-AGD and Mu-Dex-AGBD cells than in Mu-Dex-AG and Mu-Dex-AGB cells, respectively, indicating that dexamethasone promotes ALP expression in MuSCs. Osteocalcin mRNA expression in Mu-Dex cells without BMP-2 (Mu-Dex-AG and AGD cells) was significantly lower than that in Mu-Dex cells with BMP-2 treatment (Mu-Dex-AGB and AGBD cells). Among the BMP-2-treated Mu-Dex cells, Mu-Dex-AGBD cells showed significantly higher osteocalcin mRNA expression than Mu-Dex-AGB cells differentiated without dexamethasone. These findings suggest that BMP-2 plays an important role in the late stage of osteogenic differentiation of MuSCs and that its effects are enhanced by dexamethasone treatment. Thus, dexamethasone treatment throughout the culture period is critical for osteogenesis and dexamethasone enhances osteogenic differentiation with or without application of BMP-2 in both bone marrow-derived and muscle-derived stromal cells.

### 3. SMAD signaling is responsible for the effects of continuous dexamethasone treatment and combination of BMP-2 and dexamethasone

**Figure 3:**
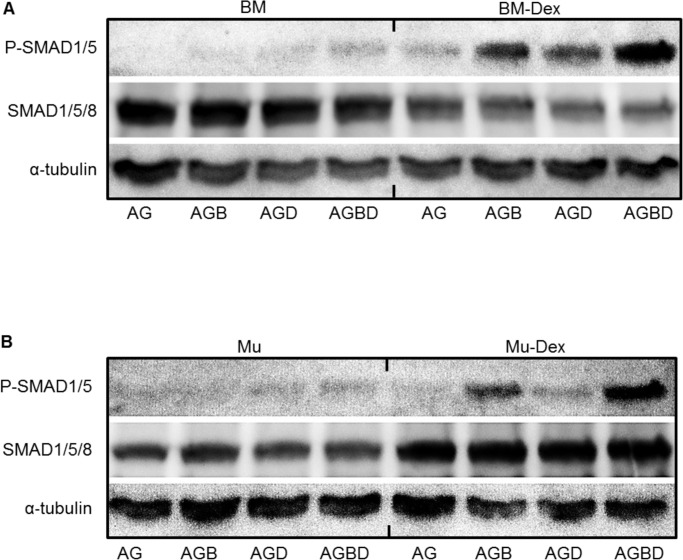
Western blot analyses of the SMAD1/5/8 and phosphorylation of SMAD 1/5. Western blot analyses of P-SMAD 1/5, SMAD1/5/8 and α-tubulin expression in BMSCs (A) and MuSCs (B) under four different osteogenic induction conditions with or without dexamethasone.

We examined BMP signaling pathways to identify the pathway responsible for the interactive effect between BMP-2 and dexamethasone. Western blot analyses of BMSCs after 24 h of osteogenic induction showed significantly increased levels of phosphorylated SMAD (P-SMAD) 1/5 in the BM-Dex cells compared to the BM cells. In the BM-Dex cells, the cells in the AGB, AGD, and AGBD conditions showed clear P-SMAD expression, and among them, the cells in the AGBD condition showed the highest P-SMAD level (Fig. 3A). There was no apparent difference in non-phosphorylated SMAD levels among the differentiation treatment groups in both BM and BM-Dex cells, although the levels in the BM cells were slightly higher than those in the BM-Dex cells. This finding indicates that the BM-Dex cells, which were expanded with continuous dexamethasone treatment, had significantly higher reactivity to BMP stimulation.

Western blot analyses of MuSCs showed results similar to those of BMSCs, i.e., increased levels of P-SMAD in the Mu-Dex-AGB and AGBD cells and a significantly higher level in Mu-Dex-AGBD cells than in Mu-Dex-AGB cells. In contrast to Mu-Dex cells, Mu-AGB and AGBD cells showed only a slight increase of P-SMAD levels. Among MuSCs, non-phosphorylated basal SMAD levels also increased in the BMP-treated groups (Fig. 3B).

Based on these results, the BMP-SMAD signaling pathway contributed to the augmentation of osteogenic differentiation in the BM-Dex and Mu-Dex groups compared with the BM and Mu groups, and further contributed to augmentation of osteogenic differentiation in the AGBD treatment groups.

### 4. Dexamethasone treatment alters the subpopulations of bone marrow-derived cells and muscle tissue-derived cells

**Figure 4:**
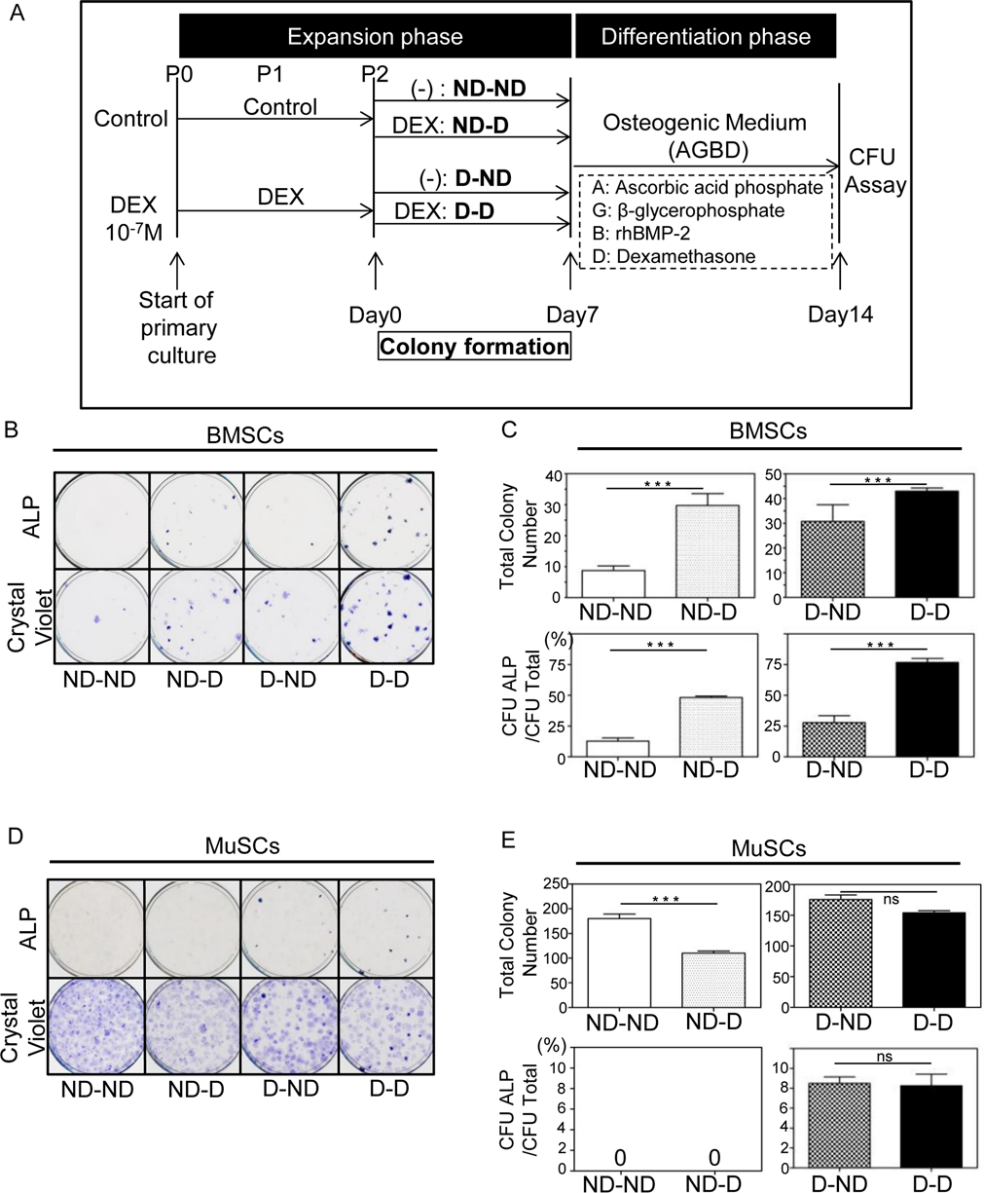
Colony formation assay of BMSCs and MuSCs. A: Schematic representation of the colony formation unit assay protocol. Cells seeded at P2 were allowed to form single-cell-derived colonies with or without 10^-7^ M dexamethasone for 7 days before osteogenic induction. ND-ND indicates normal growth medium at P0, P1, and P2. ND-D indicates normal growth medium at P0 and P1 and dexamethasone-containing medium at P2. D-ND indicates dexamethasone-containing medium at P0 and P1 and normal growth medium at P2. D-D indicates dexamethasone-containing medium at P0, P1, and P2. Gross images of BMSCs (B) and MuSCs (D) in each dish stained by ALP and crystal violet. Quantification of the total colony number and fraction of ALP-positive colonies (%) among total colonies in BMSCs (C) and MuSCs (E). *** denotes P < 0.001 as determined by Student’s t-test.

To investigate the effects of dexamethasone on subpopulations of BMSCs and MuSCs, which are both heterogeneous cell populations, colony-forming unit (CFU) assays were conducted as shown in Fig. 4A. The BM cells (ND- ), which had not been treated with dexamethasone, and BM-Dex cells (D- ), which had been exposed to dexamethasone through the culture period, were allowed to form single-cell-derived colonies with (-D) or without dexamethasone (-ND) for 7 days and then cultured in osteogenic induction medium for another 7 days. In BM cells, the ALP-positive colony ratio and total colony number were significantly higher in cultures treated with dexamethasone during colony formation (ND-D) than in those not treated with dexamethasone (ND-ND) (Fig. 4B and 4C), which indicates that dexamethasone treatment during colony formation selectively promoted the proliferation of specific subpopulations with osteogenic capability that had been contained in the BM cells and that had not been able to proliferate without dexamethasone. In BM-Dex cells, not only the ratio of ALP-positive colonies but also the total colony number was decreased by withdrawal of dexamethasone in D-ND compared to D-D, in which the cells were exposed to dexamethasone throughout the culture period (Fig. 4B and 4C). This finding indicates that some cell subpopulations that required dexamethasone to proliferate and form colonies were contained in BM-Dex cells and a part of such populations could not form colonies on withdrawal of dexamethasone. Therefore, dexamethasone may selectively promote the proliferation of cells with osteogenic potential and simultaneously suppress the proliferation of cells without differentiation potential.

Selective effects of dexamethasone were also observed in MuSCs (Fig. 4D and 4E). However, the Mu cells used in the CFU assay had been cultured in the control medium and showed extensive proliferation for 14 days without selective effects of dexamethasone. Therefore, nearly all of these cells already lacked osteogenic capability, and the ALP-positive ratio was almost 0% despite the dexamethasone treatment during colony formation. In contrast, Mu-Dex cells cultured in dexamethasone-containing medium from the beginning of the culture demonstrated an ALP-positive ratio of nearly 10% regardless of the treatment applied during the colony formation period. These results indicate that dexamethasone treatment alters the proliferation of subpopulations of BMSCs and MuSCs, resulting in an increased ratio of cells with osteogenic potential.

### 5. Dexamethasone affects cell proliferation during osteogenic differentiation

**Figure 5:**
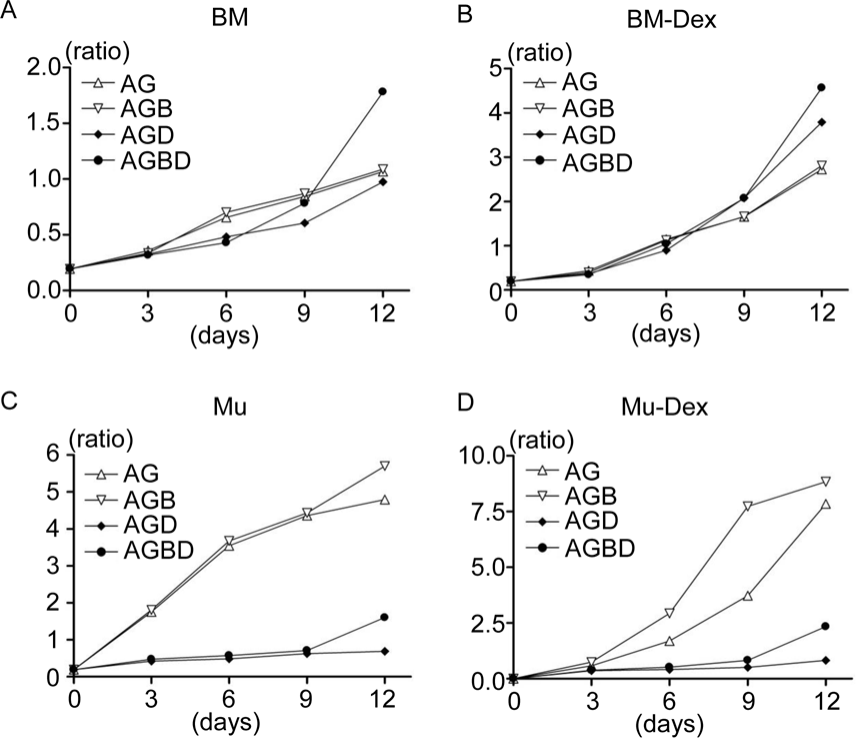
Dexamethasone affects cell proliferation during osteogenic differentiation. The absorbance at 585 nm was measured for dye extracted from the wells, and ratios relative to the standard are presented in the graphs. A: BM, B: BM-Dex, C: Mu, and D: Mu-Dex

Cell proliferation during osteogenic differentiation was analyzed by quantifying the amount of dye bound to the cells. The BM and BM-Dex cells in each osteogenic differentiation condition proliferated during differentiation. The BM cells guided to differentiate in the absence of dexamethasone (BM-AG and BM-AGB cells) proliferated faster than BM cells differentiated in the presence of dexamethasone (BM-AGD and BM-AGBD cells) in the early stage of differentiation, and then this pattern reversed as differentiation progressed (Fig. 5A). No significant differences in proliferation rates were observed among BM-Dex cells at the early stage of differentiation (Fig. 5B). However, the proliferation rates of BM-Dex-AG and BM-Dex-AGB cells declined at the later stage of differentiation relative to those of BM-Dex-AGD and AGBD cells cultured in dexamethasone.

In MuSCs, dexamethasone remarkably suppressed the proliferation of both Mu and Mu-Dex cells during osteogenic differentiation (Fig. 5C and 5D). Mu-Dex-AG and Mu-Dex-AGB cells, which showed slow proliferation in dexamethasone-containing medium during expansion culture, showed rapid proliferation in dexamethasone-free medium. These results were consistent those of the cell proliferation assay and indicated that dexamethasone also affects the proliferation and subpopulation composition of BMSCs and MuSCs during differentiation.

### 6. Bone formation capability of bone marrow and muscle-derived cells

**Figure 6:**
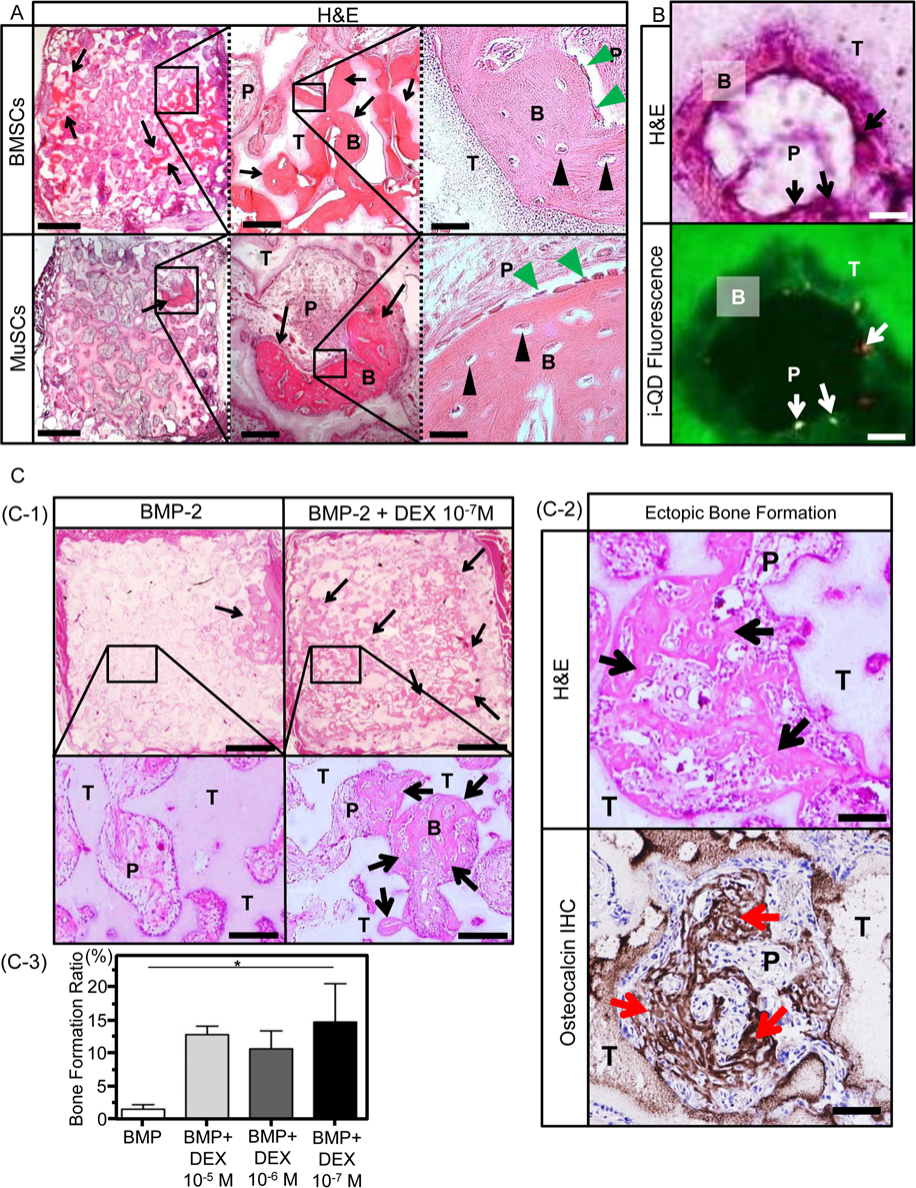
Ectopic bone formation analyses. A: Bone formation capability of muscle-derived cells. Representative histological sections of a scaffold loaded with BMSCs or MuSCs cultured with both dexamethasone and BMP-2. Scale bar: 1 mm (left panels), 200 μm (middle panels) and 50 μm (right panels). Black arrows indicate new bone formation in the scaffold. Black arrow heads indicate osteocytes and green arrow heads indicate bone lining cells. B: Newly formed bone, T: β-TCP, P: Porous area. B: Recruitment of cells residing in muscle tissue to participate in BMP-2-induced ectopic bone formation. Cells labeled prior to local BMP-2 administration were detected in the newly formed woven bone area. Representative histological sections were stained with H&E and evaluated for i-QD fluorescence. The black arrows in the H&E image show the locations of fluorescently labeled cells indicated with white arrows in the fluorescent image. Scale bar: 200 μm. B: Newly formed bone, T: β-TCP, P: Porous area. C: Augmentation of ectopic bone formation by dexamethasone. C-1, 2: Representative histological sections of an excised scaffold that had been loaded with BMP-2 alone and a scaffold that had been loaded with dexamethasone and BMP-2. The sections were stained with H&E and immunostained for osteocalcin. Black arrows indicate new bone formation in the scaffold. Red arrows indicate osteocalcin positive staining area. Scale bar: 1 mm (top panels of C-1), 200 μm (bottom panels of C-1) and 50 μm (C-2). B: Newly formed bone, T: β-TCP, P: Porous area. C-3: Quantification of bone formation at 3 weeks after transplantation. The Y axis indicates the bone formation ratio calculated as total bone area/total scaffold area. Each bar represents the mean with the standard deviation (SD). *denotes *P* < 0.05.

To confirm the bone formation capability of BMSCs and MuSCs, porous β-TCP blocks loaded with bone marrow and muscle-derived cells that had been expanded with or without dexamethasone and with or without BMP-2 were subcutaneously transplanted into rats. After 4 weeks, the implants were harvested and histologically examined (Fig. 6A). In the rats treated with BMSCs, abundant bone formation was observed in every treatment condition. In the rats treated with MuSCs, bone tissue was identified in all four blocks of the group that received cells cultured with both dexamethasone and BMP-2, although the bone tissue occupied only a small portion of each implant. No bone tissue was observed in the blocks of the other groups. This result was consistent with the in vitro mineralization results in cultured MuSCs and confirmed the bone formation capability of muscle-derived cells.

### 7. Recruitment of cells residing in muscle tissue for ectopic bone formation induced by BMP-2

It is well known that BMP-2 injected into muscle tissue induces bone formation at the administration site. To characterize the cells recruited to BMP-2-administered sites for heterotopic bone formation, i-QDs were injected into muscle tissue prior to local BMP-2 administration. Fluorescence microscopy revealed the presence of i-QD-labeled cells among bone-forming osteoblasts in the muscle tissue (Fig. 6B), which indicates that endogenous cells in the muscle tissue were recruited to participate in heterotopic bone formation induced by BMP-2.

### 8. Augmentation of ectopic bone formation by dexamethasone

Based on these results, we determined whether dexamethasone augments bone formation induced by BMP-2 in vivo. Fig. 6C-1 presents representative histological sections from an excised β-TCP block that had been loaded with BMP-2 and a block that had been loaded with both BMP-2 and 0.031 ng of dexamethasone. Histology indicated increased bone formation in β-TCP blocks that contained both dexamethasone and BMP-2 relative to blocks that contained only BMP-2, and the formed tissue was confirmed to be bone tissue by immunostaining of osteocalcin (Fig. 6C-2). The area of the formed heterotopic bone was quantified (Fig. 6C-3). There was no significant difference in the area of formed bone among the groups treated with dexamethasone; however, the area of formed bone in the groups treated with dexamethasone was significantly higher than that in the group treated with BMP-2 alone, thus confirming that dexamethasone also augments the osteogenic activity of BMP in vivo.

## Discussion

In this study, we evaluated BMSCs and MuSCs, which are thought to contribute to ectopic bone formation induced by BMPs. In both cell types, dexamethasone treatment during expansion culture resulted in considerably higher subsequent osteogenic differentiation capability. Dexamethasone treatment during osteogenic induction also promoted osteogenic differentiation induced both with and without BMP-2, and the combination of dexamethasone and BMP-2 had the strongest effect on osteogenic differentiation in both BMSCs and MuSCs.

The proliferation study and CFU assay revealed that dexamethasone differentially affected the proliferation and composition of cell subpopulations, resulting in the selection of cells with higher osteogenic capability among both BMSCs and MuSCs. Furthermore, dexamethasone treatment had a similar effect on cell proliferation during osteogenic induction, which suggests that dexamethasone also exerts selective effects on cell subpopulation composition during differentiation. Based on these results, we confirmed that subpopulation selection is at least one of the mechanisms by which dexamethasone augments the osteogenic differentiation of BMSCs and MuSCs, not only during expansion but also during osteogenic induction. The western blot analyses indicated augmented BMP-SMAD signaling in BMSCs and MuSCs expanded with dexamethasone compared to those expanded without dexamethasone. Therefore, we speculate that cells that had higher responsiveness to BMP stimulation selectively proliferated under continuous dexamethasone treatment. However, among the differentiation treatment groups, the combination of BMP and Dex in AGBD cells resulted in a significantly higher level of P-SMAD than that observed in AGB and AGD cells. This finding suggests that mechanisms other than selective proliferation may be involved in the enhanced osteogenic differentiation observed for Dex, particularly because the differences were evident within 24 h of the treatments.

Many studies using BMSCs obtained by primary culture have shown effects of dexamethasone on not only osteogenic differentiation but also chondrogenic and adipogenic differentiation. However, the mechanisms underlying these effects have not been clarified. Some studies have evaluated the effects of dexamethasone on osteoblastic or progenitor cell lines established from not only rodents but also humans. However, most of these studies aimed to clarify the mechanisms underlying steroid-induced osteoporosis [38–44]; furthermore, the cell lines evaluated were homogenous and the findings may thus be difficult to generalize to physiologically or clinically relevant cell populations. Dexamethasone has not been used for osteogenic induction in most cell lines, even though it has been shown that dexamethasone is required for osteogenic induction of heterogeneous bone marrow-derived cells and cells from other stromal tissues. Therefore, we focused on the effects of dexamethasone on heterogeneous BMSC populations. The findings in this study agreed with our previous findings that human BMSCs treated with dexamethasone during proliferation presented enhanced osteogenic, chondrogenic, and adipogenic differentiation [29]. To date, many studies have characterized the osteogenesis of BMSCs using dexamethasone. However, most of those studies did not account for the heterogeneity of the cells, and few reports have directly indicated a selective effect of dexamethasone on BMSC subpopulations such as that shown in the present study. Moreover, this is the first study to report effects of dexamethasone on MuSC proliferation. Aubin and colleagues extensively investigated the effects of dexamethasone on various types of stromal cells. They reported that dexamethasone redistributed the subpopulations of fetal rat calvaria-derived cells, resulting in enhanced osteogenic [45], chondrogenic [46], and adipogenic differentiation [47]. They also studied effects of dexamethasone on subpopulations of rat BMSCs and demonstrated that dexamethasone significantly increased subpopulations with ALP positivity and bone nodule formation [48]. Furthermore, they studied the effects of dexamethasone on rat BMSCs and reported that BMSCs contained various subpopulations. BMSCs at the early stages of culture contained subpopulations that formed bone nodules without dexamethasone treatment, and such subpopulations decreased as the culture duration increased. Dexamethasone altered the composition of the subpopulations and increased the subpopulations of BMSCs that required dexamethasone to form bone nodules. Furthermore, Aubin and colleagues indicated that some subpopulations contained in BMSCs inhibited osteogenic differentiation and that dexamethasone may affect differentiation indirectly through such subpopulations [49]. Some reports have also indicated that the timing of dexamethasone treatment is important, i.e., that treatment at the early stage of primary culture has a stronger effect on osteogenic differentiation [50]. Such results also support a selective effect of dexamethasone on cell subpopulations, as the results were obtained during the early proliferative phase, when cell selection by competitive proliferation is likely to occur. However, as mentioned above regarding the western blot analysis, such population-selective effects of dexamethasone cannot completely explain the observed enhancement of osteogenesis. In this regard, we have also confirmed that dexamethasone augments the osteogenic differentiation of immortalized human BMSCs, which are considered to be a single-cell-derived and homogenous population because they have been passaged numerous times (data not published). Furthermore, Mikami et al. previously reported a synergistic effect of dexamethasone and BMP-2 in the C3H10T1/2 cell line [51]. These studies using homogenous cell populations may thus depict a different process than our proposed mechanism of cell subpopulation selection by competitive proliferation, which can only be studied in a heterogeneous cell population such as that introduced by the present study. Although we could not completely clarify the effects of dexamethasone on the osteogenesis of stromal cells, we succeeded in demonstrating the subpopulation selection effect of dexamethasone, which can only be evaluated in heterogeneous cell populations, in addition to the dexamethasone-mediated augmentation of osteogenic differentiation induced by BMP-2. We consider that our results in heterogeneous cells obtained by primary culture are more applicable to in vivo conditions and clinical bone regeneration.

Regarding heterotopic bone formation induced by BMP-2 in muscle tissue, BMSCs, which have high osteogenic capability, and MuSCs, which reside around the implants, are expected to contribute to bone formation. Otsuru et al. directly confirmed that circulating bone marrow-derived progenitor cells differentiated into osteoblasts and formed bone tissue at muscle sites that received BMP-2-containing scaffolds in a mouse parabiotic pairing model [52]. Several previous studies demonstrated the contribution of muscle-derived cells to BMP-induced ectopic bone formation by transplanting muscle tissue-derived cells combined with BMP-2 into muscle [53, 54]. We also evaluated whether MuSCs contribute to heterotopic bone formation by BMP-2 and have bone formation capability. To identify the cells responsible for the ectopic bone formation, we labeled the cells in the muscle compartment recipient site prior to implantation. Fluorescently labeled cells were found at the site of ectopic bone formation, indicating that the labeled muscle cells migrated into the scaffold and differentiated into osteoblasts. We also performed a cell transplantation experiment to further evaluate the bone formation capability of MuSCs. Only MuSCs cultured with dexamethasone and BMP-2 showed bone formation in subcutaneous sites, although the area of the formed bone was limited. This finding simultaneously confirmed the bone formation capability of MuSCs and the effectiveness of the combination of dexamethasone and BMP-2 for MuSC-derived osteogenesis.

Previously, it was reported that systemic administration of dexamethasone enhanced ectopic bone formation by BMP-7 in murine muscles. In particular, implantation of a dexamethasone pellet into subcutaneous tissue increased the volume of ectopic bone induced by BMP-7 by 102% at 20 days of BMP-7 application and increased osteoblast number and osteoblast surface area in the ectopic bone without affecting osteoclast activity. At least during the experimental period, implantation of the dexamethasone pellet did not affect bone volume, trabecular thickness, osteoblast number, and osteoblast surface area of vertebrae and tibiae [55], which suggests that dexamethasone only affects osteoblast precursor or stem cells residing at sites with strong osteoblast induction such as those treated with BMPs. In our study, the scaffold-mediated delivery of dexamethasone and BMP-2 strongly promoted ectopic bone formation relative to BMP-2 alone, and this study is thus the first to show that local delivery of dexamethasone enhances the osteogenic effect of BMP-2 in vivo. Based on the results of our in vitro studies and in vivo studies, we speculate that both migrating muscle cells and circulating bone marrow-derived cells may have been exposed to the dexamethasone and BMP-2 in the scaffolds, resulting in their differentiation into osteoblasts to form ectopic bone. We used porous β-TCP blocks as a carrier for BMP-2 and dexamethasone and also as a scaffold for bone formation. It is well known that BMPs strongly adsorb onto calcium phosphate materials including β-TCP. Therefore, we consider that β-TCP is an appropriate carrier material for BMPs. However, we confirmed that dexamethasone does not adsorb onto β-TCP (data not shown) and may not be an appropriate carrier material for dexamethasone. Recently, the efficacy of dexamethasone-loaded CMCht/PAMAM dendrimer nanoparticles to enhance internalization of dexamethasone and subsequent osteoblastic differentiation of BMSCs was reported [56, 57]. Therefore, the use of dexamethasone loaded in such a carrier may further enhance the bone formation induced by BMP-2.

BMPs were approved for use in spine surgery by the US Food and Drug Administration late in 2002. Since then, BMP2 has been widely used not only for spinal fusion but also in other surgeries that require strong osteoinduction. However, side effects such as heterotopic bone formation [10–13], postoperative inflammation [12, 14–17], osteolysis and subsidence of implants [18–21], and cyst-like bone void formation [18] are of concern [22] and may result from excessive dosing of BMP-2. To overcome these negative aspects, many studies have attempted to develop BMP carriers to provide controlled release [58–62], whereas other studies have attempted to enhance the osteogenic inductivity of BMPs by combining them with other drugs or cytokines in vitro and in vivo [62–64]. However the findings of these trials have not yet been widely applied in the clinic. In the present study, addition of a small amount of dexamethasone to BMP-2 markedly augmented bone formation. Although dexamethasone has also been associated with complications such as osteoporosis and immunosuppression, the doses of dexamethasone used to augment bone formation in the present study were very low. Therefore, combined use of dexamethasone and BMPs may reduce the amount of BMPs required to achieve clinical efficacy, thus reducing both the cost of the procedure and also the side effect profile.

This study is not without limitations. Although we confirmed that the subpopulation selection effects of dexamethasone enhanced the differentiation capabilities of BMSCs and MuSCs, we have yet to specifically characterize the different cells in the heterogeneous cell population or to reveal the mechanism of subpopulation selection. Additionally, although the combination of dexamethasone and BMP-2 augmented bone formation both in vivo and in vitro in a rat model, it is well known that the response to BMPs is different among animal species. Therefore, for clinical applications, the quantities of dexamethasone and BMPs should be optimized.

## Conclusion

This is the first study to show that the combination of BMP-2 and dexamethasone augments the osteogenic differentiation of both BMSCs and MuSCs. We also demonstrated a strong effect of the combination of BMP-2 and dexamethasone on ectopic bone formation in vivo. These data suggest that dexamethasone could be used clinically to augment the effects of BMP-2 on bone formation. Further studies to elucidate the underlying mechanisms are required.
